# Generation of Nutrients and Detoxification: Possible Roles of Yeasts in Leaf-Cutting Ant Nests

**DOI:** 10.3390/insects3010228

**Published:** 2012-02-17

**Authors:** Thais D. Mendes, André Rodrigues, Ifeloju Dayo-Owoyemi, Fernando A. L. Marson, Fernando C. Pagnocca

**Affiliations:** 1 EMBRAPA—Agroenergy/Parque Estação Biológica, Brasília, DF 70770-901, Brazil; E-Mail: thais.demarchi@embrapa.br; 2 Department of Biochemistry and Microbiology, UNESP—São Paulo State University, Rio Claro, SP 13506-900, Brazil; E-Mails: andrer@rc.unesp.br (A.R.); newblueroses@yahoo.com (I.D.-O.); 3 Centre for the Study of Social Insects, UNESP—São Paulo State University, Rio Claro, SP 13506-900, Brazil; E-Mail: bdmarson@uol.com.br

**Keywords:** fungi, Attini, metabolism, polysaccharidases

## Abstract

The possible roles played by yeasts in attine ant nests are mostly unknown. Here we present our investigations on the plant polysaccharide degradation profile of 82 yeasts isolated from fungus gardens of *Atta* and *Acromyrmex* species to demonstrate that yeasts found in ant nests may play the role of making nutrients readily available throughout the garden and detoxification of compounds that may be deleterious to the ants and their fungal cultivar. Among the yeasts screened, 65% exhibited cellulolytic enzymes, 44% exhibited pectinolytic activity while 27% and 17% possess enzyme systems for the degradation of protease and amylase, respectively. Galacturonic acid, which had been reported in previous work to be poorly assimilated by the ant fungus and also to have a negative effect on ants’ survival, was assimilated by 64% and 79% of yeasts isolated from nests of *A. texana* and *Acromyrmex* respectively. Our results suggest that yeasts found in ant nests may participate in generation of nutrients and removal of potentially toxic compounds, thereby contributing to the stability of the complex microbiota found in the leaf-cutting ant nests.

## 1. Introduction

Ants in the tribe Attini maintain a mutualistic association with basidiomycetous fungi cultivated for food [1]. The phylogenetically derived genera in this tribe, *Atta* and *Acromyrmex*, known as the leaf-cutting ants, cut and collect fresh leaves and flower parts as substrate for the cultivation of mutualistic fungi [1,2]. This fungus is responsible for the production of extracellular enzymes that help to breakdown the plant substrate found in the fungus gardens [3,4,5,6]. In this process, simple sugars and other nutrients are generated and accumulated in the fungus gardens and it is thought these may in part support development of workers [7].

Because ants provide an optimum environment for fungal growth, additional alien microbes may also find the fungus garden a suitable substrate for development. Thus, it is fundamental for the ants to protect the mutualistic fungus from potential harmful microorganisms. For this reason ants employ several mechanisms such as: (i) massive inoculation of the cultivar onto fresh collected plant substrate [8]; (ii) weeding and grooming behaviors to remove harmful fungi [9]; (iii) secretion of antimicrobial compounds by workers [10,11,12,13,14]; (iv) the use of antibiotic-producing actinobacteria [15,16,17,18,19].

Despite the hygienic strategies adopted by ants to keep their gardens free of alien microbes, members of the fungal genus *Escovopsis *are frequently found in attine gardens. *Escovopsis* is considered a specialized parasite that attacks the cultivar hyphae [20] and long-term infections occasionally drive the colony to death [2]. Furthermore, colonies infected with *Escovopsis* show lesser garden biomass and lower number of workers and brood than uninfected colonies, thus indicating the negative effects of this parasite in the symbiosis [2]. In addition to *Escovopsis*, several other microorganisms are found in this symbiosis [21,22,23,24,25,26,27,28,29,30,31,32,33,34]. It is likely that many microbes found in the fungus gardens may not play significant roles, but some may provide unknown but important functions.

Several authors have reported the occurrence of yeasts in attine gardens. Craven *et al*. [22] studied the fungus garden of *Atta cephalotes *and* Acromyrmex octospinosus* and reported the first occurrence of yeasts in attine ant nests. Angelis *et al*. [23] isolated yeasts in gardens of *Atta sexdens* and *Atta laevigata*. Carreiro *et al*. [24] isolated the yeast genera *Candida*, *Cryptococcus*, *Rhodotorula* and *Trichosporon *from laboratory nests of *Atta sexdens rubropilosa*. Black yeasts were also isolated from different parts of the ant’s integument [25,26,27]. Rodrigues *et al*. [28] isolated yeasts from fungus garden of *A. texana* and observed inhibition of the growth of *Escovopsis* and alien filamentous fungi, suggesting that they may also be involved in defending the fungus garden. In addition, three new yeast species namely *Sympodiomyces attinorum* [32], *Cryptococcus haglerorum *[33] and *Trichosporon chiarellii *[34] were described from this environment.

Preliminary studies suggest that bacteria may act as co-participants with the mutualistic fungus, in the degradation of the plant substrate. Ribeiro [29] and Bacci *et al*. [30] isolated bacteria from fungus garden of *Atta sexdens* and found strains that exhibited cellulases, pectinases, amylases and proteases. Recently, Suen *et al*. [35] revealed that the fungus garden contain a diverse community of bacteria with high lignocellulose-degrading capacity. Pinto-Tomás *et al*. [36] reported that bacteria in the fungus garden may fix nitrogen and it seems to be an important contribution for the ants. The genus *Klebsiella* was found as one of the most effective in this task.

To investigate the potential of yeasts as co-participants in the degradation of the plant material, we carried out a preliminary screening of cellulase, pectinase, amylase, xylanase and protease enzymes exhibited by yeasts associated with fungus gardens of leaf-cutting ants. By assessing the enzymatic profile of garden yeasts, we can begin to determine the putative contribution of these microorganisms in the degradation of the main plant polysaccharides, protein material and in the assimilation of its hydrolysis products. We show that the yeast community found in fungus gardens may play more important roles than previously thought.

## 2. Experimental Section

### 2.1. Yeast Isolates and Identification

We screened a total of 82 yeasts recovered from eight leaf-cutting ant species. Forty-four yeast strains were isolated and identified by Rodrigues *et al*. [28] from the fungus garden of *Atta texana *in Texas, USA. In addition, 38 isolates were recovered from 18 nests of several *Acromyrmex* species namely *Acromyrmex ambiguus *(n = 3 nests), *Ac. coronatus *(2), *Ac. disciger *(1), *Ac. heyeri *(3), *Ac. laticeps *(4), *Ac. lundi *(3), *Ac. subterraneus *(1)and *Acromyrmex *sp. (1) collected in south Brazil (Table S1). Samples from fungus gardens of *Acromyrmex* were processed as indicated in Rodrigues *et al*. [28] but using YMA medium without rose bengal as growth restrictor.

Yeasts isolated from *Acromyrmex* were first grouped based on phenotypic characteristics. Then, genomic DNA was extracted according to Almeida [37] and subjected to microsatellite-primed PCR (MSP-PCR) with primer (GTG)_5_, following the method of Sampaio *et al*. [38]. The D1/D2 domains of 26S rDNA of representatives strains (Table S2) were amplified using primers NL1 and NL4 [39]. Reactions were composed of 8.3 μL of Milli-Q water, 2.0 μL of each primer (10 µM), 2.5 μL of 10× buffer, 1.0 μL of MgCl_2_ (50 mM), 4.0 μL of dNTPs (1.25 mM each), 0.2 μL of Taq polymerase (5 U/μL) and 5.0 μL of diluted DNA templates (1:750). The reaction conditions were: 96 °C for 3 min, followed by 35 cycles at 96 °C for 30 s, 61 °C for 45 s and final extension at 72 °C for 1 min. Amplicons were purified with the *Illustra GFX PCR DNA and Gel Band Purification Kit* (GE Healthcare). Forward and reverse sequences were generated using the same amplification primers and using the BigDye Terminator kit on ABI 377 and ABI 3100 (Life Technologies).

Sequences were assembled in contigs and manually edited using BioEdit v.7.0.5.3 [40]. Contigs were queried at the NCBI-GenBank database (National Center for Biotechnology Information) using BLASTn algorithm [41]. Sequences derived from yeast isolated from *Acromyrmex* were deposited at GenBank (JQ317161-JQ317168). Sequence accessions for yeasts isolated from *A. texana* are provided in [28].

### 2.2. Assaying Yeasts for Hydrolytic Enzymes

**Figure S1:**
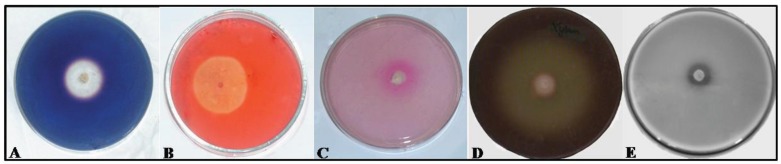
Polymer hydrolysis assays. Figure shows zones of hydrolysis of the tested polymers: (**A**) Amylase, produced by *Pseudozyma *sp. (ATT068); (**B**) CMCellulase, produced by *Trichosporon montevideense *(88c1); (**C**) Pectinase, produced by *Pseudozyma *sp. (ATT068); (**D**) Xylanase, produced by *Cryptococcus *cf. *cellulolyticus* (ATT064) and (**E**) Protease, produced by *Pseudozyma *sp. (ATT068).

Screenings for cellulase, pectinase, amylase, xylanase and protease enzymes were carried out qualitatively on different media supplemented with a specific polymer as described below. All plates were incubated at 25 °C for seven days for the detection of amylase, protease, and pectinase and 7–14 days for cellulase and xylanase. The formation of hydrolysis halos around the colonies indicated the production of enzymes and consequent degradation of polymers (see Figure S1 for representative examples of halo formation).

Starch hydrolysis was evaluated using the basal medium I described by Looder [42] supplemented with 20 g·L^−1^of soluble starch (Mallinckrodt). After incubation, plates were stained with lugol. For the assessment of CMCellulase, pectinase and xylanase a basal medium composed of 6.7 g·L^−1^ of YNB (Yeast Nitrogen Base, Difco) and 18 g·L^−1^ of agar supplemented with 5 g·L^−1^ of carboxymethylcellulose, 10 g·L^−1^ of polygalacturonic acid or 10 g·L^−1^ of xylan was used, respectively. The plates were stained with Congo red for CMCellulase [43], ruthenium red for pectinase [44] and lugol for xylanase [45]. Protein hydrolysis was assessed in solid medium containing 100 g·L^−1^ of Skim Milk medium (Difco). All polymers were supplied by Sigma Chemical Company.

### 2.3. Assimilation of the Hydrolysis Products

In addition to the hydrolytic enzymes, we evaluated the ability of yeasts to assimilate the simple sugars and products resultant of plant polysaccharides hydrolysis: maltose, cellobiose, galacturonic acid and xylose (Sigma Chemical Company). The determination was carried out on solid media containing 6.7 g·L^−1^ of YNB and 18 g·L^−1^ of agar supplemented with 5 g·L^−1^ of each carbon source. The growth of colonies on the simple sugars was compared with negative (medium without any carbon source) and positive controls (medium supplemented with glucose).

## 3. Results and Discussion

Several studies reported the occurrence of yeasts in the fungus gardens of both lower and higher attine ants [22,23,24,25,26,27,28,32,33,34,46]. Yeasts may enter the fungus gardens by the foraging activity of workers; specifically for leaf-cutting ants, yeasts may derive from the fresh plant material collected by workers as the phylloplane is considered a rich source of yeast species [47].

**Figure 1:**
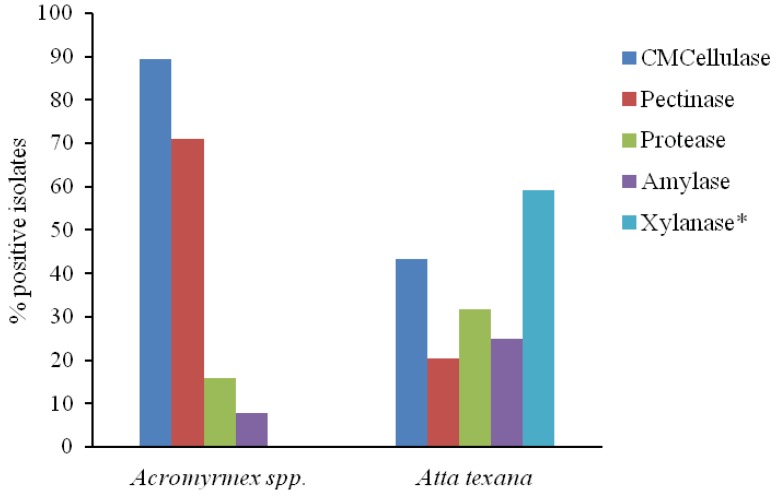
Proportion of hydrolytic yeast isolates recovered from fungus gardens of the leaf-cutting ant genera *Acromyrmex* (n = 38 yeast isolates) and *Atta* (n = 44 isolates). For details check Table 1 and Table 2. Xylanase activity was not determinate for yeasts recovered from *Acromyrmex*.

Degradation profile and assimilation of hydrolysis products by 82 yeasts isolated from *Atta texana* and *Acromyrmex* are shown in Table 1 and Table 2, respectively. Specifically for yeasts isolated from fungus gardens of *A. texana*, 77% showed at least one of the studied enzymes. Among the positive strains, six exhibited only one enzyme whereas the three *Cryptococcus laurentii* isolates exhibited four enzymes (Table 1 and Table S2 for results of specific isolates). *Cryptococcus flavus* and *Pseudozyma* sp. were the only isolates positive for all enzymes evaluated (Table 1 and Table S2 for results of specific isolates). Xylanase was exhibited by 59% of the isolates, amylase, CMCellulase, pectinase and protease activities were observed in 25%, 43%, 20% and 32% of the isolates, respectively (Figure 1). All the tested yeast isolates assimilated maltose, cellobiose and xylose and 63% assimilated galacturonic acid (Table 1).

Except for xylanolytic activity, which was not evaluated for yeasts isolated from *Acromyrmex*, all isolates exhibited at least one of the evaluated enzymes (Table 2). Amylase, CMCellulase, pectinase and protease activities were observed in 8%, 89%, 71% and 16% of the isolates, respectively (Figure 1). All yeasts isolated from *Acromyrmex* assimilated xylose and approximately 61%, 61% and 79% assimilated maltose, cellobiose and galacturonic acid, respectively (Table 2).

Our results indicated that the yeast species composition differed between gardens of *A. texana* and the various *Acromyrmex* (Table 1 and Table 2). *Cryptococcus *was the only genus shared by both ant genera. This yeast genus is widely distributed in the environment and has been shown to be prevalent among the yeast communities found on the phylloplane [47,48,49], but is also commonly found in soils from grasslands, pastures, and tundra [49]. Because of its widespread occurrence, *Cryptococcus* is expected to be in contact with workers and be transported to nests through the plant material collected by workers [24]. Previous studies on attine ants also recorded this genus as member of the microbiota of attine ants [24,25,33,34,46].

**Table 1:** Enzymatic activity and assimilation profile of yeasts and yeast-like fungi isolated from fungus gardens of *Atta texana.*

Yeast Species ^3^	N ^4^	Hydrolytic Enzymes ^1^						Assimilation ^2^			
		AM	CEL	P	XYL	PRT		M	C	A	X
*Aureobasidium pullulans*	3	-	3 ^5^	-	3	2		3	3	2	3
*Bullera sinensis*	1	1	1	-	1	-		1	1	1	1
*Bulleromyces albus*	1	-	1	-	1	-		1	1	1	1
*Candida membranifaciens*	3	-	-	-	-	-		3	3	-	3
*Candida melibiosica*	1	-	-	-	-	-		1	1	-	1
*Cryptococcus laurentii*	3	3	2	1	2	-		3	3	3	3
*Cryptococcus flavus*	3	3	3	1	3	2		3	3	3	3
*Cryptococcus magnus*	3	-	3	-	2	2		3	3	-	3
*Cryptococcus luteolus*	1	-	-	-	-	1		1	1	1	1
*Cryptococcus podzolicus*	1	1	-	-	1	-		1	1	1	1
*Cryptococcus flavescens*	1	-	-	-	1	-		1	1	1	1
*Cryptococcus *cf.* cellulolyticus*	2	-	2	-	2	-		2	2	2	2
*Cryptococcus* sp. (ATT178)	1	-	-	-	1	-		1	1	1	1
*Cryptococcus* sp. 1 (ATT079)	1	-	1	-	1	-		1	1	-	1
*Cryptococcus* sp. 2 (ATT080)	1	-	-	-	-	-		1	1	-	1
*Cryptococcus* sp. 3 (ATT123)	1	-	-	-	-	-		1	1	1	1
*Cryptococcus* sp. 4 (ATT176)	1	1	-	1	1	-		1	1	1	1
*Farysizyma *sp. ^6^	1	-	-	-	1	1		1	1	1	1
*Hannaella kunmingensis* ^6^	1	-	-	-	-	-		1	1	1	1
*Kodamaea ohmeri *	1	-	-	-	-	-		1	1	-	1
*Pseudozyma* sp.	1	1	1	1	1	1		1	1	1	1
*Rhodotorula nothofagi*	1	1	-	1	1	-		1	1	-	1
*Rhodotorula mucilaginosa*	1	-	-	1	-	-		1	1	1	1
*Rhodotorula * *lactosa*	1	-	-	-	-	-		1	1	1	1
*Rhodotorula* sp. HB 1211	1	-	-	-	-	-		1	1	1	1
*Sporidiobolus ruineniae*	2	-	-	2	-	-		2	2	-	2
*Sporisorium penniseti *(yeast-like)	2	-	-	1	1	2		2	2	2	2
*Sympodiomycopsis paphiopedili*	2	-	1	-	2	1		2	2	-	2
unidentified yeast-like fungus	2	-	1	-	1	2		2	2	2	2
Total	44	11	19	9	26	14		44	44	28	44

^1^ AM: Amylase; CEL: CMCellulase; P: pectinase; XYL: xylanase; PRT: protease; -: negative results; ^2^ M: maltose; C: cellobiose; A: galacturonic acid; X: xylose; -: negative results; ^3^ GenBank accession were provided in Rodrigues *et al*. [28]; ^4^ N: Total number of yeast isolates in each species; ^5^ Figures indicate the number of positive isolates for a particular test; ^6^ These strains were previously identified in Rodrigues *et al*. [28] as *Rhodotorula cf. taiwaniana* and *Cryptococcus *cf. *luteolus* and now are confirmed to belong to *Farysizyma* sp. and *H. kunmingensis*, respectively.

**Table 2:** Enzymatic activity and assimilation profile of yeasts isolated from fungus gardens of *Acromyrmex*.

Yeast Species	N ^4^	Closest Relative ^1^			Hydrolytic Enzymes ^2^					Assimilation ^3^			
		%	Accession		AM	CEL	P	PRT		M	C	A	X
*Cryptococcus laurentii*	1	100	JQ317168		-	-	-	1 ^5^		1	1	1	1
*Galactomyces* *candidus*	15	100	JQ317161		-	15	12	2		0	0	14	15
*Meyerozyma caribbica*	1	100	JQ317162		-	1	-	-		1	1	1	1
*Meyerozyma guilliermondii*	6	100	JQ317164		-	6	6	1		6	6	3	6
*Trichosporon chiarellii*	6	100	JQ317165		-	3	4	1		6	6	6	6
*Trichosporon montevideense*	6	100	JQ317167		1	6	2	1		6	6	3	6
*Trichosporon multisporum*	3	100	JQ317166		2	3	3	-		3	3	2	3
Total	38				3	34	27	6		23	23	30	38

^1^ According to BLASTn (NCBI-GenBank) results; %: Percent identity to sequences deposited at GenBank; ^2^ AM: Amylase; CEL: CMCellulase; P: pectinase; XYL: xylanase; PRT: protease; -: negative results; ^3^ M: maltose; C: cellobiose; A: galacturonic acid; X: xylose; -: negative results; ^4^ N: Total number of yeast isolates in each species; ^5^ Figures indicate the number of positive isolates for a particular test.

Interestingly, *Trichosporon chiarellii *[34] was found in three nests, one of *Ac. heyeri *and two of *Ac. lundi *located at different sites (Table S1). This species was described from fungus gardens of *Myrmicocrypta camargoi*, a recently described lower attine [50]. So far, there are no reports of the isolation of *T. chiarellii* from sources other than attine gardens.

Although differences between the yeast communities from the target ant genera were evident, the degradation profile of plant polysaccharides by yeasts was similar between the two communities (Figure 1). In nature, yeasts are associated with specific habitats [51]; one might expect that their presence and abundance in gardens of attine ants may not be coincidental. The acidic pH and nutrient availability [52] may allow some yeast species to be selected and multiply in attine gardens. Thus, species found in such environment rich in readily available nutrients, may not only use the nutrients for their development, but also to exhibit enzymes that aid in the hydrolysis of the plant tissues, resulting in increased nutrient availability for the microbiota and facilitating substrate colonization by the mutualistic fungus.

Pectinolytic enzymes seem to play a key role in the fungus gardens because the mutualistic fungus of leaf-cutting ants exhibit such enzymes in large quantities [3,4]. The mutualistic fungus of *Atta sexdens rubropilosa* exhibits pectinase 7 times higher than amylase and more than 24 times higher than xylanase and 38 times higher than CMCellulase [3]. Pectinolytic enzymes are important because they promote the degradation of the pectic substances present in the middle lamella of plant cells, facilitating the colonization of the plant substrate and access to other cell wall components [53]. This seems to explain the large amounts of pectinolytic enzymes exhibited by the mutualistic fungus.

The proportion of yeasts that degrade pectin was 20% and 71% of isolates recovered from *A. texana* and *Acromyrmex*, respectively (Table 1 and Table 2). This would result in a further increase in pectin breakdown in the garden. However, despite the large amount of pectinolytic enzymes exhibited by the fungus cultivar, it does not present good growth in the presence of galacturonic acid, the most important hydrolysis product of pectin [3]. In addition, Silva *et al*. [7] experimentally demonstrated that galacturonic acid does not contribute to the nutrition of ants, but rather decrease the survival of *Atta sexdens *workers. Thus, it is expected that galacturonic acid would accumulate in the fungus gardens. In contrast to what was observed for the mutualistic fungus and workers, galacturonic acid was assimilated by 64% (Table 1) and 79% (Table 2) of the yeasts isolated from nests of *A. texana* and *Acromyrmex*, respectively. In addition, Carreiro [54] working with fungus gardens and refuse dumps of laboratory nests of *A. sexdens rubropilosa* recovered a very different yeast community profile when compared to our findings. However, considering these distinct communities, 22% of the isolates (n = 93 yeasts) found in Carreiro’s study also assimilated galacturonic acid. Thus, the results of two independent studies suggest that yeasts may perform a detoxification (filtering) process in attine gardens by consuming galacturonic acid. It was also observed that the assimilation of galacturonic acid was not restricted to a specific group of yeast; instead, this ability was distributed among the majority of the taxa (Table 1, Table 2 and Table S2). Thus, the utilization of galacturonic acid may be an advantage for the survival of yeasts recently introduced into the gardens and at same time it would be a remarkable contribution to the maintenance of the nest homeostasis.

An additional possible contribution of yeasts is the degradation of cellulose. This polysaccharide is the most abundant component of plant cell wall [55]. It was formerly assumed that cellulose was the main source of carbohydrates for the ant cultivar [56]. This assumption was reinforced by Bacci *et al*. [57], who showed that the mutualistic fungus exhibits cellulolytic enzymes. However, further studies demonstrated that the ability of the mutualistic fungus to degrade cellulose is limited when compared with other polysaccharidases as pectinases, amylases and xylanases [3,4]. In addition, Abril and Bucher [58] suggested that the mutualistic fungus does not exhibit cellulase, showing that the cellulolytic activity by the cultivar does not seem to be relevant. Recently, Suen and colleagues [35] determined the amount of cellulose present in the fungus gardens. The authors discovered low amounts of cellulose in mature garden parts when compared to younger portions where newly plant material is added by the ants, suggesting that cellulose degradation does occur in the fungus gardens. Using cultivation-independent techniques, the authors also showed that the fungus garden harbors a community of cellulose-degrading bacteria [35].

Our data shows that yeasts can also contribute to the degradation of cellulose present in the fungus gardens. The CMCellulose was degraded by 43% and 89% of the yeasts isolated from *A. texana* and *Acromyrmex*, respectively (Table 1 and Table 2). These data indicate that yeasts associated with fungus gardens may contribute, along with cellulose-degrading bacteria [35], to the production of easily assimilated sugars, especially glucose, for other members of the symbiosis, and also facilitate the colonization of plant tissues by the mutualistic fungus. On the other hand, cellobiose, the disaccharide resulting from the hydrolysis of cellulose, was assimilated by all yeast isolates from *Atta texana *(Table 1) and by 61% of yeasts from *Acromyrmex *(Table 2),revealing that this sugar may support the growth of yeasts. Also, there is evidence that *Atta sexdens* workers cannot utilize this sugar for survival [7] and the mutualistic fungus only grows at an intermediate rate on this carbon source [3]. Thus, cellobiose resulting from cellulose breakdown would be available for yeasts, thereby providing for their survival without competing with the other members of the symbiosis as also observed in respect to galacturonic acid.

The yeast participation in the generation of assimilable compounds from starch seems to be as important as that observed for the other plant polysaccharides. We found that 25% of the yeasts isolated from gardens of *A. texana* exhibited amylase, while only 8% of yeasts isolated from *Acromyrmex* exhibited this enzyme. The hydrolysis products of starch are mainly glucose and maltose. Glucose is the carbon source that best supports the development of the mutualistic fungus [3] and the survival of workers of *Atta sexdens *[7]. According to Silva *et al*. [59] glucose resultant from the degradation of starch by the ant cultivar is present in the fungus garden. Although the proportion of yeast capable of starch degradation is not high, they may also contribute to the hydrolysis of starch in gardens, generating easily assimilable sugars for their growth and the other members of the symbiosis. On the other hand, except for *Galactomyces candidus *(differentiated from *G. geotrichum* by growth at 35 °C according to [60]) all yeast species assimilated maltose (Table 1 and Table 2). These data indicate that the degradation of starch is essential for the maintenance of workers [7,59] and is also important for the yeasts present in nests.

Few studies have explored the use of nitrogen sources by the ants and their associated microbes in comparison to carbon sources. Abril and Bucher [61] reported that the ant cultivar grows on inorganic sources of nitrogen, but does not grow in media containing protein (peptone) as a nitrogen source. In addition, Silva *et al*. [7] reported that workers of *Atta sexdens* are not able to use peptone and suggest that ants are not able to feed on plant proteins. Recently, Pinto-Tomás *et al*. [36] observed nitrogen enrichment in the fungus gardens of attine ants. In the present study, the production of proteolytic enzymes was observed in 32% and 16% of the yeasts isolated from *A. texana* and *Acromyrmex*, respectively. The community of yeasts and the proteolytic bacteria associated with nests [29,30] could contribute to the degradation of proteins present in the plant material and generate available nitrogen, which together with the nitrogen incorporated by the action of nitrogen-fixing bacteria [36] would support the development of the mutualistic fungus, ants and other microbes.

Xylanolytic enzymes were exhibited by 59% of the yeasts isolated from *A. texana *gardens. Xylanase is the second most abundant enzyme exhibited by the mutualistic fungus [3]. Both ants and the cultivar may have mutual development supported by xylose [3,7], the hydrolysis product of xylan. Xylan-degrading yeasts may contribute to the degradation of hemicellulose present in the plant material used by the fungal cultivar. Moreover, all yeast isolates screened in the present study were able to assimilate xylose (Table 1 and Table 2), suggesting that this carbon source would also be important in the nutrition of yeasts.

It seems that the degradation of polymers present in the fungus gardens is the result of a complementary action of enzymes exhibited by the mutualistic fungus, bacteria and also yeasts that are found in the fungus garden. Such microbial consortium would generate readily available nutrients that may help to sustain the homeostasis of the attine ant-microbe symbiosis.

## 4. Conclusion

Our results demonstrated that yeasts found in attine gardens exhibit hydrolytic enzymes capable of breaking down the plant polysaccharides found in the substrate used to culture the mutualistic fungus. In addition, we showed that yeasts are able to grow on most of the oligosaccharides derived from the digestion of the plant polysaccharides. Thus, the observed enzymatic capacity of yeasts would contribute to the ant nest by generating readily available nutrients for their own growth or for the growth of other organisms involved in the symbiosis. Consequently, by doing so, yeasts may contribute to the detoxification of compounds such as galacturonic acid that is potentially harmful to the ants and not assimilated by the mutualistic fungus.

## Supplemental Material

**Table S1:** Yeast and yeast-like strains examined.

Strain Code	Yeast Species	Nest ID	Ant Species	City/State/Country ^1^	Nest Location ^2^
8a	*Galactomyces candidus*	AOMB100904-04	*Acromyrmex heyeri*	Sentinela do Sul/RS/Brazil	S 30º37'09''; W 51º33'18''
10a	*Meyerozyma guilliermondii*	AOMB120904-05	*Acromyrmex subterraneus*	Santana da Boa Vista/RS/Brazil	S 31º19'35''; W 52º44'40''
15a	*Galactomyces candidus*				
16a1	*Meyerozyma guilliermondii*	AOMB130904-02	*Acromyrmex coronatus*	Vacaria/RS/Brazil	S 28º27'51''; W 50º53'07''
16a2	*Meyerozyma guilliermondii*				
24a	*Galactomyces candidus*	AOMB040904-02	*Acromyrmex coronatus*	Piraquara/PR/Brazil	S 25º25'50''; W 49º04'56''
27a	*Galactomyces candidus*	AOMB110904-04	*Acromyrmex lundi*	Chuvisca/RS/Brazil	S 30º50'10''; W 51º55'10''
27a1	*Galactomyces candidus*				
27b1	*Trichosporon chiarellii*				
27c1	*Trichosporon chiarellii*				
27c2’	*Cryptococcus laurentii*				
28b2	*Galactomyces candidus*	AOMB110904-05	*Acromyrmex lundi*	Chuvisca/RS/Brazil	S 30º50'10''; W 51º55'10''
30b	*Trichosporon chiarellii*	AOMB110904-03	*Acromyrmex heyeri*	Chuvisca/RS/Brazil	S 30º50'10''; W 51º55'10''
30c	*Trichosporon chiarellii*				
31a	*Galactomyces candidus*	AOMB120904-09	*Acromyrmex* sp.	Santana da Boa Vista/RS/Brazil	S 30º56'40''; W 53º05'10''
31b	*Trichosporon multisporum*				
31b2	*Trichosporon montevideense*				
31c	*Galactomyces candidus*				
32a	*Trichosporon multisporum*	AOMB120904-03	*Acromyrmex ambiguus*	Santana da Boa Vista/RS/Brazil	S 31º19'35''; W 52º44'40''
32c	*Trichosporon multisporum*				
39a	*Meyerozyma caribbica*	AOMB140904-03	*Acromyrmex disciger*	Blumenau/SC/Brazil	S 26º54'04''; W 49º10'51''
39b	*Galactomyces candidus*				
56b	*Galactomyces candidus*	AOMB140904-05	*Acromyrmex laticeps*	Blumenau/SC/Brazil	S 26º54'04''; W 49º10'51''
59a	*Galactomyces candidus*	AOMB100904-07	*Acromyrmex heyeri*	Sentinela do Sul/RS/Brazil	S 30º37'09''; W 51º33'18''
59b	*Galactomyces candidus*				
63a	*Trichosporon chiarellii*	AOMB110904-11	*Acromyrmex lundi*	Chuvisca/RS/Brazil	S 30º50'10''; W 51º55'10''
63c	*Trichosporon chiarellii*				
77b	*Trichosporon montevideense*	AOMB110904-20	*Acromyrmex laticeps*	Chuvisca/RS/Brazil	S 30º50'10''; W 51º55'10''
78a	*Meyerozyma guilliermondii*	AOMB130904-10	*Acromyrmex laticeps*	Lages/SC/Brazil	-
78b	*Meyerozyma guilliermondii*				
78b2	*Meyerozyma guilliermondii*				
79b	*Galactomyces candidus*	AOMB120904-01	*Acromyrmex ambiguus*	Santana da Boa Vista/RS/Brazil	S 31º19'35''; W 52º44'40''
87a	*Galactomyces candidus*	AOMB110904-10	*Acromyrmex laticeps*	Chuvisca/RS/Brazil	S 30º50'10''; W 51º55'10''
88a1	*Trichosporon montevideense*	AOMB060904-03	*Acromyrmex ambiguus*	Nova Petrópolis/RS/Brazil	S 29º23'18''; W 50º54'40''
88a2	*Trichosporon montevideense*				
88b	*Trichosporon montevideense*				
88b2	*Galactomyces candidus*				
88c1	*Trichosporon montevideense*				
ATT001	*Candida membranifaciens*	UGM051218-02	*Atta texana*	Bastrop County/TX/USA	N 30°05'49''; W 97°13'29''
ATT002	*Candida membranifaciens*				
ATT004	*Candida membranifaciens*				
ATT003	*Candida melibiosica*				
ATT175	unidentified yeast-like fungus				
ATT176	*Cryptococcus* sp. 4				
ATT177	*Rhodotorula nothofagi*				
ATT178	*Cryptococcus* sp.				
ATT203	unidentified yeast-like fungus				
ATT204	*Cryptococcus podzolicus*				
ATT205	*Kodamaea ohmeri *				
ATT252	*Rhodotorula mucilaginosa*				
ATT253	*Cryptococcus laurentii*				
ATT254	*Sporidiobolus ruineniae*				
ATT256	*Sporidiobolus ruineniae*				
ATT255	*Sporisorium penniseti* (yeast-like)				
ATT257	*Sporisorium penniseti* (yeast-like)				
ATT258	*Rhodotorula lactosa*				
ATT259	*Cryptococcus flavus*				
ATT064	*Cryptococcus *cf. *cellulolyticus*	UGM060121-02	*Atta texana*	Austin/TX/USA	N 30°13'56.40"; W 97°39'10.80"
ATT067	*Cryptococcus *cf. *cellulolyticus*				
ATT068	*Pseudozyma* sp.				
ATT069	*Cryptococcus magnus*				
ATT070	*Farysizyma *sp. ^3^				
ATT121	*Cryptococcus magnus*				
ATT122	*Cryptococcus luteolus*				
ATT123	*Cryptococcus* sp. 3				
ATT147	*Rhodotorula* sp. HB 1211				
ATT148	*Cryptococcus magnus*				
ATT260	*Cryptococcus flavus*				
ATT262	*Aureobasidium pullulans*				
ATT263	*Aureobasidium pullulans*				
ATT269	*Aureobasidium pullulans*				
ATT073	*Cryptococcus laurentii*	UGM060121-01	*Atta texana*	Austin/TX/USA	N 30°13'58.38"; W 97°39'06.06"
ATT075	*Cryptococcus laurentii*				
ATT074	*Bullera sinensis*				
ATT078	*Bulleromyces albus*				
ATT079	*Cryptococcus* sp. 1				
ATT080	*Cryptococcus* sp. 2				
ATT082	*Hannaella kunmingensis * ^3^				
ATT120	*Cryptococcus flavescens*				
ATT264	*Sympodiomycopsis paphiopedili*				
ATT268	*Cryptococcus flavus*				
ATT271	*Sympodiomycopsis paphiopedili*				

^1^ PR: Paraná; RS: Rio Grande do Sul; SC: Santa Catarina; TX: Texas; ^2^ GPS data unit: dd mm ss and dd mm ss.ss; ^3^ These strains were previously identified in Rodrigues *et al*. [28] as *Rhodotorula *cf*. taiwaniana* and *Cryptococcus *cf. *luteolus* and now are confirmed to belong to *Farysizyma* sp. and *H. kunmingensis*, respectively.

**Table S2:** Enzymatic activity and assimilation of compounds by yeast and yeast-like fungi examined in this study.

Strain Code	Yeast Species	Hydrolytic Enzymes ^1^						Assimilation ^2^			
		AM	CEL	P	XYL	PRT		M	C	A	X
27c2’	*Cryptococcus laurentii* ^4^	-	-	-	ND ^3^	+		+	+	+	+
8a	*Galactomyces candidus* ^4^	-	+	+	ND	-		-	-	+	+
15a	*Galactomyces candidus* ^4^	-	+	+	ND	+		-	-	+	+
24a	*Galactomyces candidus*	-	+	+	ND	-		-	-	+	+
27a	*Galactomyces candidus*	-	+	+	ND	-		-	-	+	+
27a1	*Galactomyces candidus*	-	+	+	ND	-		-	-	+	+
28b2	*Galactomyces candidus*	-	+	+	ND	-		-	-	+	+
31a	*Galactomyces candidus*	-	+	+	ND	-		-	-	+	+
31c	*Galactomyces candidus*	-	+	+	ND	-		-	-	+	+
39b	*Galactomyces candidus*	-	+	+	ND	-		-	-	+	+
56b	*Galactomyces candidus*	-	+	-	ND	-		-	-	+	+
59a	*Galactomyces candidus*	-	+	+	ND	-		-	-	+	+
59b	*Galactomyces candidus*	-	+	+	ND	-		-	-	+	+
79b	*Galactomyces candidus*	-	+	+	ND	+		-	-	+	+
87a	*Galactomyces candidus*	-	+	-	ND	-		-	-	+	+
88b2	*Galactomyces candidus*	-	+	-	ND	-		-	-	-	+
39a	*Meyerozyma caribbica* ^4^	-	+	-	ND	-		+	+	+	+
10a	*Meyerozyma guilliermondii*	-	+	+	ND	-		+	+	+	+
16a1	*Meyerozyma guilliermondii* ^4^	-	+	+	ND	-		+	+	-	+
16a2	*Meyerozyma guilliermondii*	-	+	+	ND	-		+	+	-	+
78a	*Meyerozyma guilliermondii*	-	+	+	ND	+		+	+	+	+
78b	*Meyerozyma guilliermondii*	-	+	+	ND	-		+	+	+	+
78b2	*Meyerozyma guilliermondii*	-	+	+	ND	-		+	+	-	+
27b1	*Trichosporon chiarellii*	-	-	+	ND	-		+	+	+	+
27c1	*Trichosporon chiarellii*	-	+	-	ND	-		+	+	+	+
30b	*Trichosporon chiarellii*	-	-	+	ND	-		+	+	+	+
30c	*Trichosporon chiarellii* ^4^	-	-	+	ND	-		+	+	+	+
63a	*Trichosporon chiarellii*	-	+	+	ND	+		+	+	+	+
63c	*Trichosporon chiarellii*	-	+	-	ND	-		+	+	+	+
31b2	*Trichosporon montevideense*	-	+	+	ND	+		+	+	+	+
77b	*Trichosporon montevideense*	+	+	+	ND	-		+	+	+	+
88a1	*Trichosporon montevideense*	-	+	-	ND	-		+	+	-	+
88a2	*Trichosporon montevideense*	-	+	-	ND	-		+	+	-	+
88b	*Trichosporon montevideense*	-	+	-	ND	-		+	+	-	+
88c1	*Trichosporon montevideense* ^4^	-	+	-	ND	-		+	+	+	+
31b	*Trichosporon multisporum* ^4^	+	+	+	ND	-		+	+	+	+
32a	*Trichosporon multisporum*	+	+	+	ND	-		+	+	-	+
32c	*Trichosporon multisporum*	-	+	+	ND	-		+	+	+	+
ATT262	*Aureobasidium pullulans*	-	+	-	+	-		+	+	+	+
ATT263	*Aureobasidium pullulans*	-	+	-	+	+		+	+	+	+
ATT269	*Aureobasidium pullulans*	-	+	-	+	+		+	+	-	+
ATT003	*Candida melibiosica*	-	-	-	-	-		+	+	-	+
ATT001	*Candida membranifaciens*	-	-	-	-	-		+	+	-	+
ATT002	*Candida membranifaciens*	-	-	-	-	-		+	+	-	+
ATT004	*Candida membranifaciens*	-	-	-	-	-		+	+	-	+
ATT074	*Bullera sinensis*	+	+	-	+	-		+	+	+	+
ATT078	*Bulleromyces albus*	-	+	-	+	-		+	+	+	+
ATT064	*Cryptococcus *cf. *cellulolyticus*	-	+	-	+	-		+	+	+	+
ATT067	*Cryptococcus *cf. *cellulolyticus*	-	+	-	+	-		+	+	+	+
ATT259	*Cryptococcus flavus*	+	+	-	+	+		+	+	+	+
ATT260	*Cryptococcus flavus*	+	+	-	+	+		+	+	+	+
ATT268	*Cryptococcus flavus*	+	+	+	+	-		+	+	+	+
ATT120	*Cryptococcus flavescens*	-	-	-	+	-		+	+	+	+
ATT253	*Cryptococcus laurentii*	+	-	+	+	-		+	+	+	+
ATT073	*Cryptococcus laurentii*	+	+	-	+	-		+	+	+	+
ATT075	*Cryptococcus laurentii*	+	+	-	-	-		+	+	+	+
ATT122	*Cryptococcus luteolus*	-	-	-	-	+		+	+	+	+
ATT069	*Cryptococcus magnus*	-	+	-	+	+		+	+	-	+
ATT148	*Cryptococcus magnus*	-	+	-	-	+		+	+	-	+
ATT121	*Cryptococcus magnus*	-	+	-	+	-		+	+	-	+
ATT204	*Cryptococcus podzolicus*	+	-	-	+	-		+	+	+	+
ATT178	*Cryptococcus* sp.	-	-	-	+	-		+	+	+	+
ATT079	*Cryptococcus* sp. 1	-	+	-	+	-		+	+	-	+
ATT080	*Cryptococcus* sp. 2	-	-	-	-	-		+	+	-	+
ATT123	*Cryptococcus* sp. 3	-	-	-	-	-		+	+	+	+
ATT176	*Cryptococcus* sp. 4	+	-	+	+	-		+	+	+	+
ATT070	*Farysizyma *sp.	-	-	-	+	+		+	+	+	+
ATT082	*Hannaella kunmingensis*	-	-	-	-	-		+	+	+	+
ATT205	*Kodamaea ohmeri*	-	-	-	-	-		+	+	-	+
ATT068	*Pseudozyma* sp.	+	+	+	+	+		+	+	+	+
ATT258	*Rhodotorula lactose*	-	-	-	-	-		+	+	+	+
ATT252	*Rhodotorula mucilaginosa*	-	-	+	-	-		+	+	+	+
ATT177	*Rhodotorula nothofagi*	+	-	+	+	-		+	+	-	+
ATT147	*Rhodotorula* sp. HB 1211	-	-	-	-	-		+	+	+	+
ATT254	*Sporidiobolus ruineniae*	-	-	+	-	-		+	+	-	+
ATT256	*Sporidiobolus ruineniae*	-	-	+	-	-		+	+	-	+
ATT255	*Sporisorium penniseti* (yeast-like)	-	-	+	+	+		+	+	+	+
ATT257	*Sporisorium penniseti* (yeast-like)	-	-	-	-	+		+	+	+	+
ATT264	*Sympodiomycopsis paphiopedili*	-	+	-	+	-		+	+	-	+
ATT271	*Sympodiomycopsis paphiopedili*	-	-	-	+	+		+	+	-	+
ATT175	unidentified yeast-like fungus	-	+	-	+	+		+	+	+	+
ATT203	unidentified yeast-like fungus	-	-	-	-	+		+	+	+	+

^1^ AM: Amylase; CEL: CMCellulase; P: pectinase; XYL: xylanase; PRT: protease; ^2^ M: maltose; C: cellobiose; A: galacturonic acid; X: xylose; ^3^ ND: xylanase was not determined for yeasts isolated from *Acromyrmex *spp.; ^4^ Strains selected as representative for DNA sequencing.
